# Myxoma of the renal pelvis masquerading pelviureteric stenosis: conservative limited resection with renal preservation: case presentation and literature review

**DOI:** 10.1186/s12894-020-00657-8

**Published:** 2020-06-30

**Authors:** Najla Aldaoud, Amer Hallak, Liqa A. Rousan, Omar Halalsheh, Bashar Darayseh, Mousa A. Al-Abbadi

**Affiliations:** 1 Department of Pathology and Microbiology, Jordan University of Science and Technology, P.O. Box 3030, Irbid, 22110 Jordan; 2 Faculty of Medicine, Jordan University of Science and Technology, Irbid, Jordan; 3 Department of Diagnostic and Interventional Radiology and Nuclear Medicine, Jordan University of Science and Technology, Irbid, Jordan; 4 Department of Surgery and Urology, Jordan University of Science and Technology, Irbid, Jordan; 5 Department of Pathology, Microbiology and Forensic Medicine, School of Medicine, University of Jordan, Amman, Jordan

**Keywords:** Myxoma, Renal pelvis, Sinus, Hydronephrosis

## Abstract

**Background:**

Myxoma is a relatively rare mesenchymal tumor seen mainly in the heart and skin. Renal myxomas in particular are exceptionally rare where only 17 cases were previously reported in the English Language literature. Only 2 of the 17 reported cases were located in the renal sinus/pelvis.

**Case presentation:**

This is a case of an 18-year-old male patient who complained of right, colicky flank pain associated with abdominal pain and discomfort. Imaging findings revealed right kidney hydronephrosis with a provisional diagnosis of pelviureteric junction (PUJ) stenosis. On computed tomography, there was a very faint thin walled mass abutting the calyces, camouflaged within the dilated renal pelvis. During surgery, a polypoid mass was found at the pelviureteric junction, causing the obstruction. Histological examination showed a hypocellular, paucivascular myxoid neoplasm, with few spindle cells displaying serpentine nuclei and inconspicuous nucleoli. The tumor cells expressed immunoreactivity for vimentin, but not for S100, CD34, actin, or desmin. This will qualify as the third case of renal pelvis myxoma.

**Conclusion:**

Myxomas in the renal pelvis/sinus are extremely rare and can present with hydronephrosis and subtle radiological findings mimicking a PUJ stenosis. Being aware of this entity can save the patient unnecessary nephrectomy with possible preservation of the kidney.

## Background

Tumors in the renal pelvis account for around 5–10% of all renal tumors. Urothelial cell carcinoma contributes to around 90% of these cases [1]. Mesenchymal neoplasms are relatively infrequent, but mostly originate from vascular or smooth muscle tissue. Mesenchymal renal neoplasms in the renal pelvis tend to be benign, while in the ureter they are more frequently malignant [2].

Myxomas are unusual soft tissue neoplasms with predilection for the heart, soft tissues, skin, and bone. Large skeletal muscles of the thigh and buttocks have been frequently involved [3]. Nevertheless, only 17 reported cases of pure myxomas have been previously identified within the renal system in the English Language literature [3–20]. Renal myxomas do not have specific radiological findings, and they are usually misdiagnosed as malignant neoplasms. Most of these renal myxomas are located in the renal parenchyma, while those encountered in the renal sinus or capsule are extremely rare.

It is crucial to distinguish between this benign entity and malignant tumors that may show secondary myxoid changes in order to avoid overtreatment; especially with small lesions that can be treated with limited and minimally invasive surgery.

## Case presentation

An 18-year-old male patient presented with right, colicky flank pain associated with abdominal pain and discomfort for a few weeks prior to admission. Symptoms were not accompanied by fever, chills, rigors, or any lower urinary tract complaints. His physical examination was normal, apart from slight right flank tenderness with no evidence of palpable masses. All laboratory investigations were within normal limits.

**Fig. 1:**
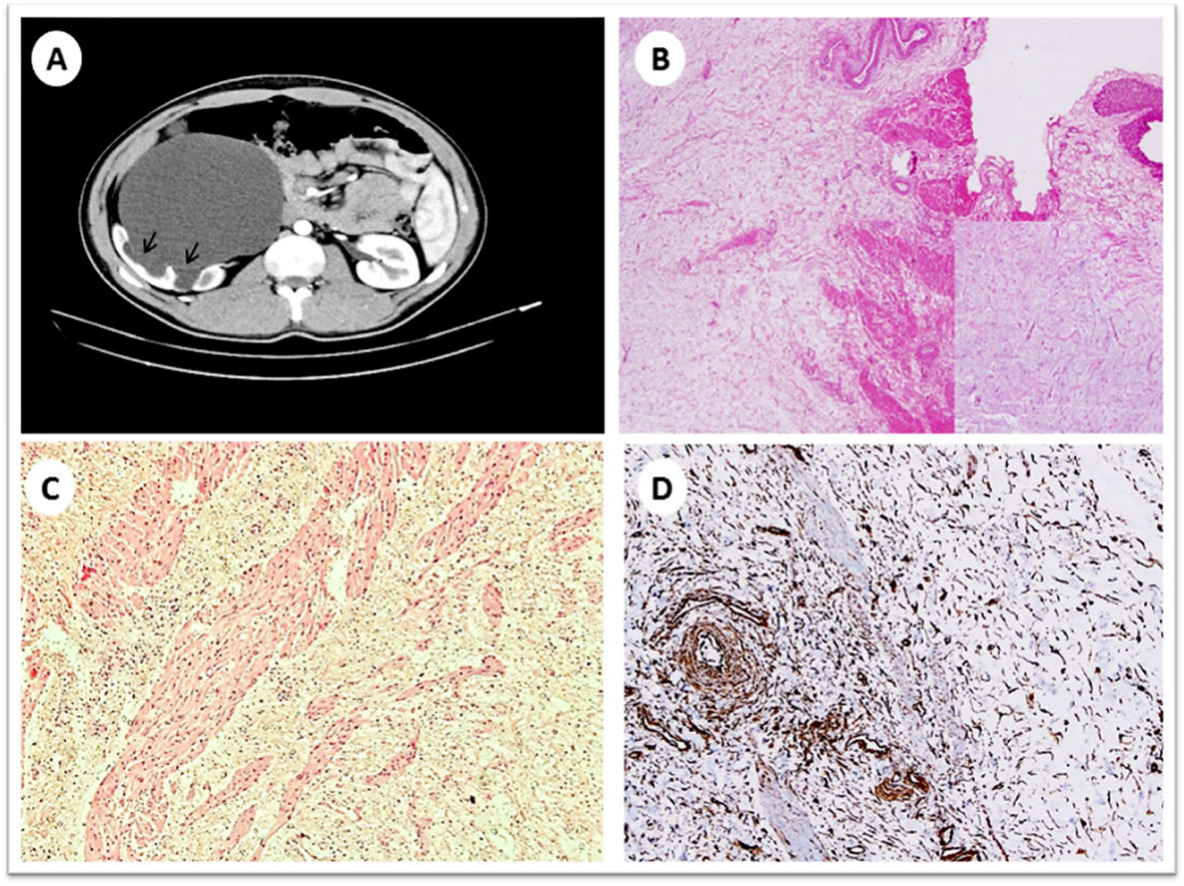
Radiological and histopathological images for the myxoma case. **a** Enhanced computed tomography scan shows severe right sided hydronephrosis with ballooning of the renal pelvis and thinning of the renal cortex, a faint thin curvilinear wall seen abutting the dilated calyces (arrows). **b** Low power view of an ill-defined myxoid lesion with overlying unremarkable urothelium at the upper right (Hematoxylin and eosin 40X); the insert shows a medium power of spindle / stellate cells with no atypia and a myxoid background. **c** Low power view showing infiltration of the tumor into the muscularis propria (Hematoxylin and eosin 40X). **d** Medium power view showing positive staining of the tumor cells for vimentin immunostain (200X)

A computed tomography urography scan showed an enlarged right kidney with severe hydronephrosis and ballooning of the renal pelvis, associated with thinning of the overlying renal cortex. The right ureter was not dilated. The density of the ballooned renal pelvis was similar to that of urine, however, there was a thin, faint curvilinear wall abutting the dilated calyces. This was suggestive of the presence of a mass occupying the renal pelvis, a finding which was overlooked during the initial imaging evaluation. Therefore, the diagnosis was suggestive of PUJ obstruction (Fig. 1).

A double J stent was temporarily inserted into the right kidney and pyeloplastic surgical intervention was deemed appropriate. Intraoperatively, a mass was felt at the PUJ which was resected and sent for frozen section evaluation. The specimen contained a portion of the renal pelvis measuring 5.5 × 2.0 × 1.0 cm. A polypoid mass was identified and measured 2 × 1.8 × 1.8 cm. The mass was 1 cm away from the proximal margin and 2 cm from the distal margin. On serial sectioning, an ill-defined mass with white, soft, gelatinous cut surface was observed. Frozen section examination showed an unremarkable urothelial lining with a subepithelial hypocellular myxoid lesion with sparse blood vessels and few spindle cells with serpentine nuclei and inconspicuous nucleoli. There was no evidence of necrosis or increased mitotic activity, therefore, a frozen section interpretation of “myxoid lesion” was given to the surgical team. Consequently, the surgical team decided to proceed with conservative resection of the mass, and save the patient unnecessary nephrectomy. The permanent histological examination revealed an ill-defined mass similar to the frozen section appearance. The tumor involved the muscularis propria but not the urothelial mucosa. No extension beyond the renal pelvis was identified (Fig. 1). By well-controlled, routine immunohistochemical stains, the tumor cells were immunoreactive for vimentin (Fig. 1), while they were negative for actin, desmin, S-100 and CD34 antibodies. Based on the tumor morphology and the immunoprofile, the final diagnosis of renal pelvis myxoma was rendered. The patient was discharged two days after surgery with no immediate complications, and he has been doing well for the past 16 months.

## Discussion and conclusions

The first myxoma to be reported in the capsule of the kidney was in 1887 by Hulk [14]. Myxoma cells are thought to originate from fibroblast-like, primitive mesenchymal cells that lack the ability to polymerize collagen [6]. The characteristic gelatinous texture on gross examination is a result of disproportionate amounts of glycosaminoglycans [6, 8].

In most of the previously reported renal cases, myxomas were incidentally detected. However, the most common symptom reported is flank pain. Hematuria, renal colic, urinary tract infection, and obstructive uropathy have also been described [3, 8]. Unfortunately, renal myxomas lack any specific radiological findings and are often mistaken for other malignant lesions, especially renal cell carcinoma. Radiologically, they present as cystic or solid masses with variable degrees of enhancement, and are more commonly located within the renal parenchyma [4, 12]. In our case, there was an ill-defined mass where a faint, thin wall was overlooked on initial imaging studies. We can only speculate that this may be due to the fact that the mass was cystic in nature; its contents had the same density of urine, so the lesion was camouflaged within the fluid attenuated, dilated renal pelvis. Unfortunately, a preoperative magnetic resonance imaging was not performed, which could have added more necessary imaging features.

Pure renal myxomas have been exceptionally unusual [4]. Only 17 reported cases of pure myxomas have been previously identified in the English language literature [3–20]. Parenchymal involvement seemed to be the most frequent location; however, only 2 of these cases involved the renal sinus and pelvis (Table 1). These two cases, reported by Appel et al. [5] and Yildirim et al. [6], showed a large, radiologically visible, renal pelvic/sinus mass. In contrast to our case, that was a small 2 cm mass with subtle radiological findings. Our case is unique because it is the smallest myxoma tumor reported in the kidney thus far. In comparison, the reported cases in the literature had a range from 4 to 28 cm [6]. Our patient is also the youngest among all cases reported, as the age range at the time of diagnosis was from 27 to 82 years [6].

**Table 1:** Clinicopathologic data of the 3 cases of renal sinus/pelvis myxoma in the English language literature

Authors	Age/Sex	Site/location	Presenting symptom	Tumor size	Treatment
Appel and Schoenberg [5]	No/No	Right parapelvic	Hematuria for 2 months	8 cm	Enucleation of mass
Yildirim et al. [6]	82/Male	Left renal sinus	Dysuria, flank pain, urinary obstruction for two years	9 cm	Nephrectomy
Our case	18/Male	Right renal sinus	Right colicky flank pain, abdominal pain, discomfort for weeks	2 × 1.8 × 1.8 cm	Resection of mass

A nephrectomy was performed in all cases except Appel et al. [5, 6], where only enucleation of the mass was performed. In our case a limited, conservative resection of the mass was performed, with preservation of the kidney.

It is essential to differentiate renal myxomas from other benign and malignant tumors that can exhibit secondary myxoid changes. These tumors include perineuroma, myxoid neurofibroma, myxoid leiomyoma, myxolipoma, leiomyosarcoma, rhabdomyosarcoma, extraskeletal chondrosarcoma, low-grade fibromyxoid sarcoma, and myxofibrosarcoma [15, 16]. Our case did not show any areas of neural, fibrous, or muscle differentiation. The absence of S-100 expression ruled out neurofibroma, chondrosarcoma, and lipoma. In addition, the tumor cells in our case didn’t express actin or desmin which helped in ruling out smooth and skeletal muscle tumors. The lack of atypia and necrosis distinguished it easily from other sarcomas with secondary myxoid changes.

There seems to be no clear consensus on the appropriate treatment of myxomas in the renal sinus. Yildirim et al. [6] stated that the treatment of choice is radical nephrectomy, but enucleation of the tumor as an alternative method has also been reported by Appel and Schoenberg [5], and is preserved for situations where there is no infiltration into renal parenchyma. As for our case, the unique extent of tumor growth and the polypoid nature of the mass at the PUJ made resection possible with preservation of the remaining collecting system.

In conclusion, Myxomas in the renal pelvis/sinus are extremely rare. Our patient presented with non-specific symptoms, hydronephrosis, and an ill-defined, vague mass in the renal pelvis. We highlight the importance of considering this rare, benign tumor and the importance of performing frozen section for suspicious lesions to avoid radical surgery with possible preservation of the kidney.

## Data Availability

The datasets used and/or analyzed during the current study are available from the corresponding author on reasonable request. All data generated or analyzed during this study are included in this published article.

## Abbreviation

PUJ: Pelviureteric junction
